# Understanding issues associated with attending a young adult diabetes clinic: a case study

**DOI:** 10.1111/j.1464-5491.2011.03447.x

**Published:** 2012-02

**Authors:** R Snow, N Fulop

**Affiliations:** NIHR King’s Patient Safety and Service Quality Research CentreKing’s College London, UK

**Keywords:** adherence, adolescence, healthcare delivery, patient experience, Type 1 diabetes

## Abstract

Diabet. Med. 29, 257–259 (2012)

**Aims:**

To study the reasons for attendance behaviour from the patient viewpoint at a young adult diabetes outpatient clinic.

**Methods:**

Attendance rates for 231 clinic appointments over 19 months for 102 patients were calculated. Semi-structured interviews were conducted with a purposive sample of 17 of the 102. The interviews encouraged participants to describe routines, thoughts and feelings around clinic appointments. Observations were made of the clinic system. Themes arising from patients’ emotional and practical issues around attendance were generated from the data.

**Results:**

**‘**Did not attend’ rates for the clinic over the study period were 15.7%. However, bureaucratic problems created many ‘missed’ appointments; most instances of ‘did not attend’ investigated were attributable to communication failures. Participants did not divide neatly into ‘attenders’/’non-attenders’; many had complex mixed attendance records. Most weighed the value of attendance against immediate obstacles such as incompatible work/clinic hours. Reminders were seen as important, particularly for this age group. Respondents identified fear of being judged for ‘poor control’ as a major factor in attendance decisions, suggesting that having a high HbA_1c_ level may lead to non-attendance, rather than vice versa.

**Conclusions:**

Health professionals’ supportive, non-judgemental attitude is important to patients considering clinic attendance. In this study, improved communication, reminders and flexible hours might reduce ‘did not attend’ rates.

## Introduction

Improving attendance rates at outpatient clinics is often seen as important both in terms of avoiding the waste of medical resources and in terms of better overall health outcomes [1]. Much of the medical literature on non-attendance in diabetes points to significantly higher HbA_1c_ results amongst ‘defaulters’ as an example of the benefits of clinic attendance [2]. In UK diabetes care, outpatient clinic attendance rates vary widely, from 75% non-attendance [3] to 1.4% [4]. There is evidence that young people miss more scheduled medical appointments of all kinds than other age groups [3,5]. Indeed, for younger patients with diabetes, the transition from paediatric to adult clinic can be crucial, with many people dropping out of the system altogether [6]. Within diabetes outpatient care, socio-demographic factors, such as gender and class, do not seem to be associated with missed appointments, although some have found single parents and smokers to be more likely not to attend [7]; patients who feel that their recommended treatment is not effective are also less likely to seek specialist care at clinic [8]. Overall, however, reviews of the existing literature do not offer conclusive reasons for non-attendance and show that clinic-related factors behind non-attendance are rarely assessed, with the patient voice largely absent from the debate [9,10]. This study aimed to help redress that balance by exploring issues around attendance for this vulnerable age group, from the patient point of view.

A specific young adult diabetes clinic was taken as an ‘exemplifying’ case study [11], to assess in depth what attendance means for those registered there. The study was led by a researcher with Type 1 diabetes. Questions centred on the value of clinic to this group of patients, the physical, emotional and practical barriers to attendance, and the processes involved in the decision to go—or not to go—to clinic.

## Patients and methods

The case study young adult clinic accepts all 18- to 25-year-olds with Type 1 diabetes within a single county in south-east England. Three types of data were collected: (1) attendance records were analysed for 231 appointments for 102 individuals from November 2008 to May 2010; (2) semi-structured interviews were carried out with 17 patients registered at the young adult clinic; (3) the appointments and cancellation telephone line was monitored over a 3-week period.

Using the data collected as described above, a purposive approach to sampling for the interview study was employed [12], with 17 participants (nine men and eight women) selected on grounds of relevance to the questions driving the research—in this case, attendance behaviour.

The interviewees included seven who were recorded as regularly attending clinic appointments, five with a record of intermittent attendance and three who had never attended within the survey period. A further two participants were chosen because they were new to the young adult clinic following extended periods of non-attendance.

The decision-making process relating to clinic attendance was used as a framework to allow participants to identify the areas they considered important. The interviews were conducted as semi-structured one-to-one discussions of 20–30 min each. Themes arising from patients’ emotional and practical issues around clinic attendance were derived from the data.

The study gained National Health Service (NHS) ethics approval under REC reference 10/H0718/1.

## Results

Patients could not be divided into ‘attenders’ and ‘non-attenders’; many showed a complex record of attendance, non-attendance and cancellations. Overall DNA (Did not attend) rates across the study period were calculated using NHS guidelines [13] at 15.7% (36 recorded DNAs/231 scheduled appointments). However, this figure should be treated with caution. Most patients had more than one scheduled appointment during the survey period, so it was possible to gather further data on 18 appointments from the patients’ perspective during the 17 interviews described above. Eleven instances recorded as ‘did not attend’ were attributable to problems with administration, communication and bureaucracy, combining to create false ‘missed’ appointments. Patients faced great difficulty accessing the central booking line and internal hospital communication problems meant that cancellations and changes of address were not always passed on to the clinic. The audit of the cancellation service showed that there could be many as 17 people waiting in the telephone queueing system at peak times and a wait of over 20 min to speak to an operator; on some occasions, the call simply disconnected with no option to wait or leave a message. In interviews, some patients mentioned that they had been warned by staff or friends not to bother with the central number, as they would not get through.

Within the study sample, participants could be grouped into those who made a cost–benefit analysis of the obstacles and benefits of going to clinic, and those who did not think about it at all; some moved from one group to another over time. In the ‘cost–benefit analysis’ group, valued benefits included practical information (in an ideal world, delivered by others with diabetes), timely test results, emotional support and reassurance.

‘You know, it’s all very well saying, oh, ‘get better control’ but it’s not always that easy… it would be helpful if there was someone who actually had diabetes that you could talk to and say oh I’m having trouble with this, what can I do with that… you could maybe fit it into the *real* world, you know, how it would work and not just in theory’. Woman, age 24, diagnosed in childhood

The value of friendly, positive reception and clinical staff was appreciated by all and a reliable system of reminders by text or email was seen by this age-group as very useful for ensuring appointments were not missed.

‘I think everyone’s on mobile and email these days aren’t they, so I think that would be better than [a] letter… You know what teenage boys and that are like. I mean I forget anything’. Man, age 23, diagnosed in childhood

For some, the clinic’s available hours were not compatible with unsympathetic employers’ demands.

Interviewer: ‘Did you have to book holiday [from your job]?’

Respondent: ‘Six weeks’ notice just for a day, and that was quite hard… if we were really busy then [the boss] would say no, you can’t have it’. Man, age 23, diagnosed in childhood

Many respondents identified that being ‘told off for poor control’ by health professionals of all kinds could be a major obstruction to future attendance at clinic.

‘They look at you really disapprovingly, and it’s like, please *don’t* because there is, you know, I’m not just doing it because I can’t be arsed… there’s obviously a reason for it so just sort of, I don’t know, not analyse it but just look to see why and don’t judge’. Woman, age 21, diagnosed in adolescence

Amongst those patients who did not think about whether or not to go to clinic, some always attended out of routine. Parents often played an important role in supporting this routine. Others went through a period of non-attendance, often referring to this afterwards as ‘denial’. This concept of a phase where the condition feels unmanageable was a common theme and may be seen as part of the normal process of chronic disease [14].

‘It’s a very emotional, I mean when you are diagnosed with something new, you know, your mind, I mean I was really, really depressed. I mean come on, who wouldn’t be, you know, it’s such a thing, and at that stage I can’t even handle most [doctors]’. Woman, age 25, diagnosed in adulthood

## Discussion

In this study, patients’ attendance behaviour was complex, with many respondents reporting a change in attitude over time. For the majority of those interviewed, their attendance record was dependent on the value offered at clinic vs. the obstacles put up by inflexible hours, bureaucratic procedures and by health professionals’ attitudes to diabetes.

In addition, information-sharing problems inflated the number of appointments recorded as ‘did not attend’; the clinic’s true non-attendance rate is likely to be considerably lower than the 15.7% initially documented. As interviewees were not selected at random, but deliberately chosen to give a range of attendance behaviours, it is not possible to give an accurate estimate of the real ‘did not attend’ rate during the survey period. However, the study found that at least 31% (11/36) of all unattended appointments could have been avoided, by improving communication between clinic and hospital trust. Even assuming the remaining uninvestigated instances of ‘did not attend’ were accurately recorded, this may bring the clinic’s true overall ‘did not attend’ rate closer to 10 or 11%.

Previous studies of non-attendance assume a causal connection between missed appointments and associated higher HbA_1c_ [2,3]. Results from this study, however, indicate that fear of being ‘told off ’ for failing to reach biomedical targets was an important factor in the decision not to attend. In other words, rather than non-attendance causing high blood glucose readings, perhaps high blood glucose readings—or health professionals’ reactions to them—cause non-attendance. Any benefits clinic may offer in terms of screening, particularly valuable to those struggling to control their diabetes, will then of course also be missed.

This study suggests two main implications for service delivery. Firstly, it may be worthwhile for clinics with apparently high ‘did not attend’ rates to conduct audits of their own booking procedures to identify where messages are going astray or where cancellation and rebooking may be particularly difficult. Secondly, the research highlights the importance of diabetes professionals’ reactions to young people’s HbA_1c_ results. Censorious responses to ‘poor’ control may in fact be contributing to patients’ decisions to stop attending clinic. In this study, an understanding of the difficulties in managing diabetes, plus timely and practical information, were among the most highly valued things health professionals could offer participants.

The research is limited in a number of ways. As with all case studies, findings cannot be reliably generalized to other clinics. In particular, the region studied is above average in terms of employment and income, with limited ethnic diversity, and the catchment area includes a highly educated university population. Regions with fewer resources, a more heterogeneous and complex pool of patients and, of course, a different age group might yield very different themes. However, although in-depth research into attendance from the patient viewpoint is rare, comparable studies of people with Type 1 diabetes have flagged up identical issues; particularly the need for flexible hours, positive emotional support and understanding from others with diabetes, and non-judgemental advice from health professionals [15,16].

There is potential for future research in similar clinics using a ‘patient-eye-view’ approach, to explore where clinics might be able to reduce obstacles and enhance the value they offer their patients in order to improve attendance rates.

## Competing interests

Nothing to declare.
